# Assessment of Knowledge and Attitude Regarding Epilepsy and Seizure First Aid Among Male Teachers in Mecca Region, Saudi Arabia: A Cross-Sectional Study

**DOI:** 10.7759/cureus.30945

**Published:** 2022-10-31

**Authors:** Hatem Alsulami, Salma Alhadhrami, Bashair Alshareef, Renad Alqurashi, Asma Alzahrani, Amal Alkhotani

**Affiliations:** 1 Department of Medicine, Faculty of Medicine, Umm Al-Qura University, Mecca, SAU

**Keywords:** first aid training, epileptic seizures, seizure firstaid, attitude, knowledge, teachers, first aid, seizure, epilepsy

## Abstract

Background: ​​Epilepsy is one of the most common neurological disorders in the pediatric age group, and teachers have a crucial role in providing appropriate epilepsy first aid. This study aims to assess the knowledge of and attitude toward epilepsy and seizure first aid among male teachers in the Mecca region, Saudi Arabia.

Methods: This cross-sectional study used an online self-administered questionnaire. A 22-item questionnaire was distributed via social media platforms between November 18, 2021, and February 15, 2022. The study used descriptive statistics to describe the participants' characteristics, and relations were tested using the Pearson chi-square test.

Results: Of 385 male teachers, approximately two-thirds (57.9%) of teachers had poor knowledge of epilepsy and its first aid, 86.5% of teachers were aware that epilepsy is a neurological disorder, 67% correctly reported that during epileptic seizures, they should ensure the patient's safety and seek help, 37.7% of teachers knew when they should transfer the students to the hospital, and 45.3% who had a positive attitude towards children with epilepsy had good knowledge regarding the disease (P=.010). Only 13% had training on how to deal with seizures.

Conclusion: Overall knowledge of epilepsy and its first aid among schoolteachers in Mecca is still inadequate. It necessitates the inclusion of a nationwide, specialized educational epilepsy program into the teacher training curriculum.

## Introduction

In any school, at any time, there is a chance for an emergency to happen. Epilepsy is one of the most common neurological disorders in the pediatric age group, with a higher rate of occurrence during the school years [1]. Recent studies indicate that the highest prevalence occurs in infants less than one year and those aged 1-12 years at a rate of 102 cases per 100,000 per year, while the incidence in children aged 11-17 years is 21-24 per 100,000 cases [2]. Childhood is the typical time of onset of many epilepsy syndromes and one of them is childhood absence seizure [3]. The prevalence of epilepsy is 6.5 per 1000 people in Saudi Arabia [4]. It has been estimated that the global prevalence rate of epilepsy is around 5.16 per 1000, based on the results of 32 studies conducted in various countries around the world [5], and it accounts for one of the highest proportions of neurological disorders in children [4]. According to International League Against Epilepsy (ILAE), epilepsy syndrome is defined as a characteristic cluster of clinical and EEG features, often accompanied by specific etiological findings (structural, genetic, metabolic, immune, and infectious) [6]. 

The school years constitute a challenging phase in a child's life. Students who are diagnosed with epilepsy are more likely to experience learning difficulties and poor academic performance, as well as social isolation, mental health issues, and low self-confidence, all of which can impair their future and life as adults [4]. Also, they often experience worry due to the unexpected nature of seizures and the possible implications of having a seizure at school [7]. Since epilepsy is common among school-aged children and since seizures can happen at school, instructors would typically be the first to administer medical attention [8]. Recent research has shown that Southern European nations like Italy and Greece have increased awareness and understanding of epilepsy [9,10]. In contrast, Asian nations like India, Jordan, and Malaysia have more unfavorable attitudes about the disease [4]. Even though teachers are the first line who witness seizures, Saudi teachers lack enough knowledge of their significant impact on the lives of students who have epilepsy at school. According to previous studies, teachers in Saudi Arabia, typically in Tabuk, Mecca, Taif, and Jeddah cities, have been shown to have insufficient understanding, inadequate training, and misconceptions about epilepsy and its management and first aid [1,4,11,12].

The development of a child's health, performance, and social skills can be significantly impacted by a teacher's understanding of epilepsy and seizure first aid [8]. Hence, before establishing and implementing a health education program, it is crucial to investigate the degree of knowledge, attitudes, and beliefs held by school instructors.

As studies in Saudi Arabia are limited, and no study has been conducted before on male teachers in the Mecca region, the present study aims to assess the knowledge and attitude toward epilepsy and seizure first aid among male teachers in the Mecca region, Saudi Arabia.

## Materials and methods

Study design

A descriptive cross-sectional study using an online self-administered questionnaire was conducted between November 18, 2021, and February 15, 2022, in the Mecca region of Saudi Arabia

Sampling strategy 

Convenience sampling techniques were used to enroll study participants. The sample included all-male teachers in Saudi Arabia who teach in Mecca schools; these inclusion criteria were displayed in the invitation letter that was sent along with the study survey link. In addition, the survey was distributed via social media platforms like Twitter (Twitter, Inc., San Francisco, California, United States), WhatsApp (Meta Platforms, Menlo Park, California, United States), and Snapchat (Snap Inc., Santa Monica, California, United States). Since all the participants volunteered to participate in the study, there was no need for written informed consent.

Questionnaire tool

The questionnaire was adapted using scales that have previously been verified [11]. It is divided into three sections and contains 22 items. Section one involved demographic information, including basic information about their type of school, years of experience, and educational level. Section two included general information about epilepsy to assess their knowledge. Finally, section three included seizure first aid, post-event care, and when a patient should be transferred to a medical facility.

Sample size 

The minimal sample size required for a prevalence study was estimated using the equation proposed by Charan and Biswas in their article titled "How to Calculate Sample Size for Different Study Designs in Medical Research?" [13]. Based on a 95% confidence level and a 5% margin of error, and a standard deviation of 0.5, the sample size required was 385 participants. 

Statistical analysis

After data were extracted, it was revised, coded, and fed to the statistical software IBM SPSS Statistics for Windows, Version 22.0 (Released 2013; IBM Corp., Armonk, New York, United States). All statistical analysis was done using two-tailed tests. A P-value of less than 0.05 was statistically significant. For knowledge and awareness items, each correct answer was scored one point, and a total summation of the discrete scores of the different items was calculated. A patient with a score of less than 60% (0-6 points) of the total score was considered to have poor awareness, while good awareness was considered if he had a score of 60% (7-10) of the total or more. Descriptive analysis based on frequency and percent distribution was done for all variables, including teachers' socio-demographic data, type of school, teaching stage, experience at teaching, and training regarding dealing with seizures. Also, teachers' knowledge and awareness regarding epilepsy and its first aid were shown in frequency tables and graphs. Cross tabulation was used to assess the distribution of teachers' overall knowledge and awareness level regarding epilepsy and its first aid according to their personal data and attitude. Relations were tested using the Pearson chi-square test and an exact probability test for small frequency distributions.

Ethical approval 

This study was approved by the Faculty of Medicine at the University of Umm Al-Qura, Mecca, Saudi Arabia (approval number HAPO-02-K-012-2021-11-823).

## Results

Demographic characteristics

A total of 385 teachers fulfilling the inclusion criteria completed the study questionnaire. Of the 385 participants, 369 (95.8%) work at governmental schools, and 16 (4.2%) work at private schools. Regarding qualification, 343 (89.1%) had a bachelor's degree, while 35 (9.1%) had a postgraduate degree. A total of 160 teachers (41.6%) taught primary-level students, 102 (26.5%) taught at an intermediate stage, and 123 (31.9%) taught secondary-level students. Regarding teaching experience, 354 (91.9%) had more than 10 years of experience, while 12 (3.1%) had an experience of one to five years. Around 128 (33.2%) teachers had a relative or friend who has epilepsy, 264 (68.6%) saw a case with an epileptic seizure, and 49 (12.7%) had training on how to deal with seizure attacks (Table 1).

**Table 1:** Participant’s Demographic Characteristics

Personal data	No	%
School type		
Governmental	369	95.8%
Private	16	4.2%
Qualification		
Diplome / institute	7	1.8%
Bachelor	343	89.1%
Master / Ph.D.	35	9.1%
Teach stage		
Primary	160	41.6%
Middle	102	26.5%
Secondary	123	31.9%
Teaching experience years		
1-5	12	3.1%
6-10	19	4.9%
> 10	354	91.9%
Has a relative or friend who has epilepsy?		
Yes	128	33.2%
No	257	66.8%
Has anyone ever had a case of epileptic seizure in front of you?		
Yes	264	68.6%
No	121	31.4%
Received any training on how to deal with seizures		
Yes	49	12.7%
No	336	87.3%

Knowledge and attitude with regard to epilepsy

**Figure 1:**
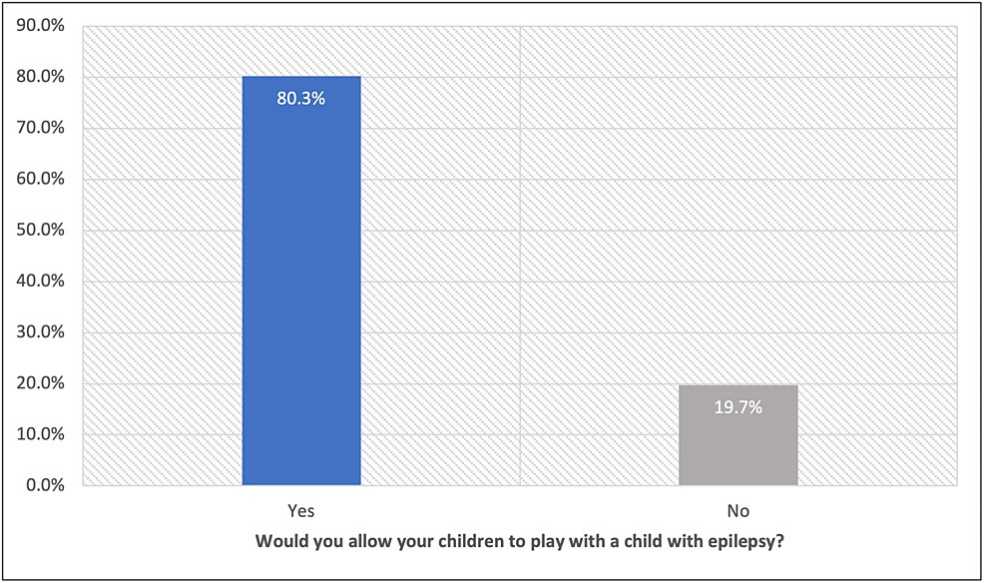
Teachers' Attitude Towards Children With Epilepsy

Of the 385 teachers who participated in the survey, 333 (86.5%) were aware that epilepsy is a neurological disease and 75.3% knew that there is a treatment for epilepsy, while only 63.4% knew that epilepsy drugs don't cause addiction. Regarding the nature of seizures, 37.1% of the teachers knew that epileptic seizures did not usually cause loss of consciousness, and only 15.3% reported that epileptic seizures might be in the form of a lack of concentration. A total of 72.2% of the teachers correctly reported that children with epilepsy could join ordinary schools, and 98.2% knew that epilepsy is not an infectious disease (Table 2). On asking teachers if they would allow their children to play with a child with epilepsy, 309 (80.3%) answered “yes” while 76 (19.7%) answered “No” (Figure 1).

**Table 2:** ‏Knowledge Regarding Epilepsy And Seizure First Aid Among Male ‏Teachers in Mecca city

Knowledge items	No	%
Epilepsy is		
Neurological disease	333	86.5%
Psychological disease	41	10.6%
Magic	11	2.9%
There is a treatment for epilepsy		
Yes	290	75.3%
No	95	24.7%
Epilepsy drugs cause addiction?		
Yes	141	36.6%
No	244	63.4%
Epileptic seizures usually cause loss of consciousness?		
Yes	242	62.9%
No	143	37.1%
Epileptic seizures may be in the form of lack of concentration		
Yes	59	15.3%
No	326	84.7%
Children with epilepsy should study at specialized school		
Yes	107	27.8%
No	278	72.2%
Epilepsy may be infectious		
Yes	7	1.8%
No	378	98.2%
Action during epileptic seizure		
Ensure the patient safety and seek help	258	67.0%
Placing the patient and trying to stop the movement	20	5.2%
He opened his mouth and put a gauze in it	107	27.8%
Action after end of seizure attack		
Put the student on his side and ask for help	225	58.4%
Attempt to wake the student	49	12.7%
Wash his face with water and give him water	93	24.2%
Read the Holy Quran	18	4.7%
When should the student be transferred to the hospital?		
Immediately with the seizure attack	33	8.6%
If the seizure lasts more than 5 minutes	31	8.1%
If the seizure lasts more than 10 minutes	25	6.5%
If the seizure lasts more than 20 minutes	14	3.6%
When the seizure is repeated without the student waking up	58	15.1%
Both 1 & 2	79	20.5%
Both 2 & 5	145	37.7%

Knowledge of seizure first aid and post-event care

Regarding the overall level of knowledge among teachers regarding epilepsy and its first aid, 162 (42.1%) teachers had an excellent level of understanding of epilepsy and related first aid, whereas 223 (57.9%) teachers had a poor level of knowledge. In terms of first aid for seizure attacks, 67% of the teachers correctly reported that during epileptic seizures, they should ensure the patient's safety and seek help. Also, 58.4% knew that they should put the student on his side and ask for help after the end of seizures. A total of 37.7% of teachers knew that they should transfer the student to the hospital if the seizure lasts more than five minutes or if the attack is repeated without the student waking up (Table 2).

Distribution of teachers’ knowledge regarding epilepsy and its first aid by their personal data and attitude

There was no significant association between any of the teachers' characteristics and their knowledge level regarding epilepsy and its first aid. Only 45.3% who had a positive attitude toward children with epilepsy had good knowledge regarding the disease compared to 28.9% of others with a negative attitude with recorded statistical significance (P=.010) (Table 3). 

**Table 3:** Distribution of Teachers’ Knowledge Regarding Epilepsy And its First Aid by Their Personal Data And Attitude *: P < 0.05 (significant); ^$^: Exact probability test

Personal data	Overall knowledge level				p-value
	Poor		Good		
	No	%	No	%	
School type					.705^$^
Governmental	213	57.7%	156	42.3%	
Private	10	62.5%	6	37.5%	
Qualification					.619^$^
Diploma	4	57.1%	3	42.9%	
Bachelor's degree	196	57.1%	147	42.9%	
Master's degree/Ph.D.	23	65.7%	12	34.3%	
Teaching stage					.610
Primary	94	58.8%	66	41.3%	
Middle	62	60.8%	40	39.2%	
Secondary	67	54.5%	56	45.5%	
Teaching experience years					.633
1-5	7	58.3%	5	41.7%	
6-10	9	47.4%	10	52.6%	
> 10	207	58.5%	147	41.5%	
Has a relative or friend who has epilepsy?					.364
Yes	70	54.7%	58	45.3%	
No	153	59.5%	104	40.5%	
Has anyone ever had a case of epileptic seizure in front of you?					.839
Yes	152	57.6%	112	42.4%	
No	71	58.7%	50	41.3%	
Received any training on how to deal with seizures					.616
Yes	30	61.2%	19	38.8%	
No	193	57.4%	143	42.6%	
Allow your children to play with a child with epilepsy?					.010^*^
Yes	169	54.7%	140	45.3%	
No	54	71.1%	22	28.9%	

## Discussion

This study evaluates knowledge and attitude toward epilepsy and seizure first aid among male ‏teachers in Mecca, Saudi Arabia. In this study, approximately two-thirds (57.9%) of teachers had poor knowledge levels regarding epilepsy and its first aid. This insufficient knowledge of epilepsy was also observed in other regions in Saudi Arabia, such as Tabuk and Arar [4,14]. In contrast, teachers in Riyadh and Khamis Mushait were found to have a high understanding of epilepsy [15,16]. Internationally, teachers' degree of epilepsy knowledge was poor in Kuwait, China, and Italy [17-19]. Although the level of knowledge is insufficient, the understanding and awareness of the etiology of epilepsy have experienced a significant change throughout time. Most recent studies have revealed that most teachers believe epilepsy is a neurological disorder [19]. Consistent with the literature, the current study found that around 86% of teachers agreed that epilepsy is a neurological disease. The percentage of our study was higher than that in previous studies done in many regions of Saudi Arabia [4,7,11]. Moreover, only 10.6% of the participants in the current study thought that the cause of epilepsy was psychological. The levels in this study are far below those in Tabuk and Taif, which found that 56% to 59% of the studied teachers believed that epilepsy was a psychological disorder [4,12].

Due to the risk of seizures happening at school, teachers have a crucial role in providing appropriate first aid. They also significantly contribute to controlling the other kids and reducing the terrifying scene [19]. During an epileptic seizure, the provider should time the attack and keep the patient in a safe position [19]. Once the attack is over, they should place the patient in a recovery position and observe the level of consciousness [19]. Unless the seizure is prolonged or the patient has recurring seizure attacks without recovering consciousness, the patient does not need to be hospitalized [19]. Our study showed that most teachers correctly reported that during epileptic seizures, they should ensure the patient's safety and seek help. However, this finding contradicts previous studies on different regions in Saudi Arabia, in which it was concluded that although most teachers are confident in their ability to assist someone suffering a seizure, many continued to describe incorrect practices or were unable to handle students having actual epileptic seizures with first aid [4,12,16]. The fact that some of the questions in our study had correct responses suggests that teachers may have tried to increase their knowledge over time. Unexpectedly, our results differ from those of a previous study on female teachers in the same region, in which only 37.3% of teachers correctly responded to the question regarding ensuring student safety [11]. This result may indicate a gender difference in teachers' knowledge. Our focus in this study was on male teachers only, Segregation of men and women in education has been part of Saudi Arabia's culture and Islamic religion. In Saudi Arabia, the Ministry of Education ensures that males and females are strictly separated in schools. Although schools are separated, they are governed by the same government and adhere to the same regulations. Regarding post-event care, the results of this study show that around two-thirds of teachers believed that placing the patient in the recovery posture after an epileptic attack was the proper action. These results reflect those of Kanjo et al., who also found that most teachers in Jeddah know how to apply post-event care [8]. One unexpected finding in our study was that only around 37% answered correctly about the indication of deciding to transfer the patient to a hospital. There are similarities between the attitudes expressed by teachers in this study and those described by other studies in Saudi Arabia [8,11], indicating this area needs to be improved.

 In our study, there was no significant association between teachers' characteristics and their knowledge level regarding epilepsy and its first aid. Similarly, Abulhamail et al. could not identify any associations between teachers' age, gender, number of years of experience, or kind of institution, and their knowledge of epilepsy [1]. According to studies, more experience and a postgraduate qualification are significant factors, and teachers with more experience and those who are more educated have a higher percentage of knowledge overall [4,12,15,20-22]. The difference in the results of the studies was likely due to most of our participants having at least 10 years of experience and most had bachelor's qualifications, which pevented us from evaluating teachers with less experience and lower educational level knowledge. Regarding attitude, our study showed that 45.3% of those with a positive attitude towards children with epilepsy had good knowledge of the disease (P=.010), This corresponds with a study in ‏Palestine that showed that higher attitude scores were associated with receiving education about epilepsy [21].

Only 13% of our study participants received training on dealing with seizures. Teachers do not get formal epilepsy education as educators throughout their preparation or employment [19]. Despite their importance, school health services are regularly ignored in Saudi Arabia [19]. Therefore, it seems that teachers in Mecca still need more training on dealing with epilepsy and seizure first aid. Prior studies have noted the importance of giving teachers training on epilepsy and its first aid [23,24]. Educational training leads to improvement in knowledge and willingness to take action while lowering unnecessary and harmful interventions [23,24]. A recent interventional study in Saudi Arabia concluded that teachers' responses to seizures and overall awareness of epilepsy significantly improved as a result of the health education program [24]. This highlights the potential advantages of including a specialized intervention program for epilepsy in the teacher training curriculum. The study recommends incorporating educational training courses for teachers to correct false epilepsy information and organizing first aid training courses to understand better how to deal with a child who is having a seizure attack. There have been no studies on the skills or expertise of teachers in handling emergency drugs. Further studies, which take these variables into account, will need to be undertaken. We also urge the organization of epilepsy public awareness campaigns to address common misconceptions about the disease and encourage positive attitudes toward children with epilepsy.

Limitations 

It was necessary to address some of the limitations of our research results. This study primarily evaluated male teachers only. In Saudi Arabia, males and females attend different schools, making it difficult to apply our findings to female teachers. Second, since the study was a cross-sectional questionnaire, no practical skills could be tested in this setting, and the responses were most likely subjective. Third, it was only conducted in the Mecca region; thus, its results may have limited generalisability.

Effective interventions are required to increase knowledge, modify attitudes about epilepsy, and address current epilepsy-related behaviors. Hence, research on these interventions should be conducted using an evidence-based approach.

## Conclusions

Our study suggests that although teacher knowledge regarding epilepsy has improved over time, the overall understanding of epilepsy and its first aid among schoolteachers in Mecca is still insufficient. The majority of teachers lack epilepsy first aid training, necessitating the inclusion of a nationwide, specialized educational epilepsy program into the teacher training curriculum.
